# miR-107 Activates ATR/Chk1 Pathway and Suppress Cervical Cancer Invasion by Targeting MCL1

**DOI:** 10.1371/journal.pone.0111860

**Published:** 2014-11-11

**Authors:** Chengyan Zhou, Gang Li, Jingjing Zhou, Na Han, Zhihui Liu, Jun Yin

**Affiliations:** 1 Development and Utilization Key Laboratory of Northeast Plant Materials, Key Laboratory of Northeast Authentic Materials Research and Development in Liaoning Province, School of Traditional Chinese Materia Medica, Shenyang Pharmaceutical University, Wenhua Road 103, Shenhe District, Shenyang, 110016, China; 2 Department of Nutrition and Food Hygiene, School of Public Health, Harbin Medical University, Baojian Road 157, Nangang District, Harbin, 150081, China; Queensland University of Technology, Australia

## Abstract

MicroRNAs (miRNAs) are a class of single-stranded, non-coding RNAs of about 22 nucleotides in length. Increasing evidence implicates miRNAs may function as oncogenes or tumor suppressors. Here we showed that miR-107 directly targeted MCL1 and activated ATR/Chk1 pathway to inhibit proliferation, migration and invasiveness of cervical cancer cells. Moreover, we found that MCL1 was frequently up-regulated in cervical cancer, and knockdown of MCL1 markedly inhibited cancer cell proliferation, migration and invasion, whereas ectopic expression of MCL1 significantly enhances these properties. The restoration of MCL1 expression can counteract the effect of miR-107 on the cancer cells. Together, miR-107 is a new regulator of MCL1, and both miR-107 and MCL1 play important roles in the pathogenesis of cervical cancer. We have therefore identified a mechanism for ATR/Chk1 pathway which involves an increase in miR-107 leading to a decrease in MCL1. Correspondingly, our results revealed that miR-107 affected ATR/Chk1 signalling and gene expression, and implicated miR-107 as a therapeutic target in human cervical cancer. We also demonstrated that taxol attenuated migration and invasion in cervical cancer cells by activating the miR-107, in which miR-107 play an important role in regulating the expression of MCL1. Elucidation of this discovered MCL1 was directly regulated by miR-107 will greatly enhance our understanding of the mechanisms responsible for cervical cancer and will provide an additional arm for the development of anticancer therapies.

## Introduction

Aberrant microRNAs (miRNAs) expression is a defining feature of human malignancy. Specific miRNAs have been identified as promoters or suppressors of metastatic progression [1], [2]. Cervical cancer has also recently been shown to be associated with an abnormal miRNA expression profile, suggesting that miRNAs might contribute to cancer development [3]. Cervical carcinoma significantly affects the health of women worldwide and currently ranks as the second leading cause of cancer mortality in women following breast cancer. Approximately 500,000 cases of cervical cancer are diagnosed per year, with nearly 45% of those resulting in death [4], [5]. Cervical cancer is a complex disease involving the abnormal expression of many oncogenes and tumor suppressor genes. Although focusing on known genes has yielded significant new information, previously unknown noncoding RNAs, such as miRNAs, may also provide insights into the biology of cervical cancer.

A number of miRNAs have been identified to regulate tumor metastasis. Among them, miR-107, belonging to the miR-103/107 family due to their identical seed sequences, is capable of inducing epithelial-to-mesenchymal transition of mammary epithelial cells, thereby fostering invasive and metastatic behaviors of cancers [6]–[8]. Myeloid cell leukemia-1 (MCL1) is an anti-apoptotic member of the Bcl-2 protein family, and its expression has been found to be induced in cells at various stages of growth and differentiation [9]. Due to its anti-apoptotic properties, MCL1 is a potential proto-oncogene. In addition, enhanced expression of MCL1 is observed in a wide range of tumors, including hepatocellular carcinoma, breast cancer, etc [10]–[13]. Growing evidence suggests that MCL1 expression levels are associated with worse clinical outcomes in various cancer types. Although the miR-107 is considered to play a key role in determining tumor properties, the regulation of MCL1 expression in cervical cancers remains largely unknown. This prompted us to further analyze the relevance of MCL1 for cervical cancer.

In this study, we investigated the role played by miR-107, a miRNA associated with cervical cancer and its interaction with the suppressor MCL1. Therefore, we determined by qRT-PCR that MCL1 was overexpressed in cervical cancer relative to adjacent normal tissues, and MCL1 was identified as a direct target of miR-107. Knockdown of MCL1 suppressed the growth and invasiveness of human cervical cancer HeLa and SiHa cells. Our results indicated that MCL1 may function as an oncogene and is a mediator of miR-107 in cervical cancer. Despite the availability of various treatment modalities, such as surgery, chemotherapy, and radiotherapy, the 5-year survival remains poor. Therefore, it is absolutely necessary to explore drugs capable of preventing and treating cervical cancer. Taxol has been found to possess antitumor effects on human lung adenocarcinoma cell line A549, human hepatocellular carcinoma cell line Bel-7402, human breast adenocarcinoma cell line MCF-7 and mouse Lewis lung carcinoma cell line *etc* [14]–[16]. Thus, in this study, taxol was undertaken to find out if taxol had any effects on proliferation, differentiation and apoptosis of cervical cancer cells, and to investigate the possible mechanism on the transcription level.

## Experimental Methods

### Ethical Statement and Subjects

Written informed consents were obtained from all subjects. The experimental protocol were reviewed and approved by the Ethical Committee of Harbin Medical University (HMU-EC-10168) and was conducted according to the principles expressed in the Declaration of Helsinki.

### Plasmid Construction

Pri-miR-107 was amplified using the primers and then cloned into the restriction sites of pcDNA3. The resulting construct pcDNA3/pri-miR-107 was confirmed by DNA sequencing. A synthesized ASO-miR-107 was as an inhibitor of miR-107. The 3′-UTR fragment of the MCL1 gene containing the predicted miR-107 binding site was amplified by PCR using the primers. PCR products were cloned into the pcDNA3/EGFP plasmid. The resulting vector was named pcDNA3/EGFP-MCL1 3′-UTR. Moreover, mutant fragment of MCL1 3′-UTR containing a mutated miR-107 binding site was amplified using PCR site-directed mutagenesis and cloned into the pcDNA3/EGFP plasmid between the same sites. All insertions were confirmed by sequencing. To construct the siR-MCL1 vector, a 70-bp double-strand fragment was obtained via an annealing reaction using two single strands. The pcDNA3 vector was used to generate a MCL1 overexpression plasmid. The full-length human MCL1 cDNA sequence was amplified using PCR from a cDNA clone vector and then cloned into the restriction sites using primers.

### Cell Culture

HeLa and SiHa cell lines were obtained from Cancer Research Center of Harbin Medical University (Harbin, China). Hela cells were maintained in RPMI1640 (GIBCO) and SiHa cells were maintained in DMEM medium, these cells were supplemented with 10% fetal bovine serum (FBS; HyClone, Logan, UT), 100 IU/ml of penicillin and 100 µg/ml of streptomycin in a humidified atmosphere of 95% air and 5% CO_2_ at 37°C.

### Human Tissue Samples

Twenty-six pairs of clinical specimens, including 26 human cervical cancer tissue sections from patients with cervical cancer and corresponding adjacent normal tissues, were obtained from the First Affiliated Hospital, Harbin Medical University. Histological all the biopsies were squamous cell carcinoma and the stages of cancer were from III. The diagnoses of these samples were verified by pathologists.

### Real-time RT-PCR Analysis

Quantitative RT-PCR was performed to detect the relative transcript levels of miR-107. Briefly, 3 µg of small RNA extracted and isolated from cells or tissue samples was reverse-transcribed to cDNA with the stem-loop reverse transcriptase primer using the M-MLV reverse transcriptase (Promega, Madison, WI). cDNA was subsequently used for the amplification of miR-107 and an endogenous control, U6 snRNA, via PCR. The MCL1 reverse transcription (RT) primer and qPCR primers were synthesized by Sangon Biotech., Inc. (Shanghai, China). PCR cycles were as follows: 94°C for 3 min followed by 40 cycles of 90°C for 30 s, 55°C for 30 s, and 74°C for 30 s. To quantify MCL1 expression, 4 µg of RNA extracted from cells or tissue samples was reverse-transcribed to cDNA using the M-MLV reverse transcriptase. PCR cycles were as follows: 94°C for 3 min followed by 40 cycles of 96°C for 30 s, 56°C for 30 s, and 70°C for 30 s. SYBR green RCR was performed in triplicate using Bio-Rad Chromo 4 System Real-Time RCR detector (Bio-Rad, Hercules, CA, USA). The relative gene expression levels were calculated. All the primers were purchased from ABI.

### Western Blotting

Western blotting was performed to determine MCL1 protein expression. All proteins were resolved on 10% SDS-denatured polyacrylamide gel and were then transferred onto a PVDF membrane. Membranes were incubated with blocking buffer for 80 min at room temperature and then incubated with an antibody against MCL1 or glyceraldehyde-3-phosphate dehydrogenase (GAPDH) with overnight at 5°C. The membranes were washed and incubated with a horseradish peroxidase (HRP)-conjugated secondary antibody (AuGCT, Inc. (Beijing, China)). Protein expression was assessed by enhanced chemiluminescence and exposure to chemiluminescent film. Lab Works Image Acquisition and Analysis Software (UVP) were used to quantitate band intensities.

### Fluorescent Reporter Assay

HeLa and SiHa cells were co-transfected with pri-miR-107 or ASO-miR-107 in a 48-well plate followed by the pcDNA3/EGFP-MCL1 3′-UTR reporter vector or pcDNA3/EGFP-MCL1 3′-UTR mutant. A separate RFP expression vector, pDsRed2-N1 (Clontech, Mountain View, CA) was used for normalization. The cells were lysed 72 h later, and the proteins were harvested. The following vectors were cotransfected into cells: those containing the EGFP reporter vector alone, with pcDNA3/primiR-107, or bearing ASO-miR-107. PcDNA3/EGFP, pcDNA3 or ASO-NC were used as control vectors. The intensities of EGFP and RFP fluorescence were detected with the F-4500 Fluorescence Spectrophotometer (Hitachi, Tokyo, Japan).

### Transfection Assay

HeLa cells (1×10^5^ cells per well) were seeded into 6-well culture plates and grown overnight. Then cells were transfected with miR-107 expression vector (pri-miR-20a, 4 µg each well) or miR-107 antisense oligonucleotides (ASO-miR-107). For SiHa cells, 1×10^6^ cells per well were seeded into 6-well culture plates. 5 µg pri-miR-107 or 500 µmol ASO-miR-107 was transfected. The plasmid pcDNA3 and the non-relative sequence (ASO-NC) were used for negative control. Transfection was performed with Lipofectamine 2000 Reagent (Invitrogen, Carlsbad, CA) according to the manufacturer's protocol. The medium was replaced with new culture medium 5h after transfection. 24 h post-transfection, cells were used for cell viability, cell colony formation and in vitro migration and invasion assays.

### Assessment of Cell Viability and Proliferative Capacity

To determine cell viability and proliferative capacity, cells were examined using the MTT and colony formation assays as described previously [17]. Cells were seeded in 96-well plates at either 1×10^5^ cells/well (HeLa cells) or 1.5×10^5^ cells/well (SiHa cells) and tested using the MTT assay at different time points. For the colony formation assay, the number of viable cell colonies was determined after 10 days (HeLa cells, SiHa cells) after inoculation of 200 cells/well in triplicate in 12-well plates. The cells were stained with crystal violet. Colony formation was quantified using the colony formation number.

### Cell Migration and Invasion Assays

For the Transwell migration assay, 1×10^5^ HeLa cells or 1.3×10^5^ SiHa cells in 200 µl of RPMI 1640 without FBS were seeded into the upper part of each Transwell chamber (pore size of 8 µm; Corning) containing a non-coated membrane. For the invasion assay, 1×10^5^ HeLa cells or 1.3×10^5^ SiHa cells were placed on the upper chamber of each insert coated with 50 µl of 2 mg/ml Matrigel growth factor, and 500 µl of RPMI 1640 with 20% FBS was added to the lower part of the chamber. After incubating for several hours, the chambers were disassembled, and the membranes were stained with a 2% crystal violet solution for 15 min and placed on a glass slide. Then, cells that had migrated across the membrane were counted in five random visual fields using a light microscope. All assays were performed three independent times in triplicate.

### Immunohistochemistry

Immunofluorescence assay was performed according to the methods described previously [18]. The sections were pretreated with microwave irradiation, blocked, and incubated using polyclonal rabbit anti-human MCL1 (Saier Biotechnology). Staining intensity was assessed was measured as previously described [18].

### Cell Cycle Assay

Cells were plated and transfected with pcDNA3-miR-107 or pcDNA3-NC on day 0, then reseeded on day 1, and harvested on day 2. Samples were fixed, stained, and measured with flow cytometry according to reported protocols.

### Flow Aytometry Analysis

Apoptosis was assessed by measuring the membrane redistribution of phosphatidylserine with an annexin V-propidium iodide apoptosis detection kit (Sigma, USA). In addition, the propidium iodide-stained LoVo cells were analysed on an EPICS ELITE ESP flow cytometer (Beckman Coulter, USA).

### Taxol Regulates Expression of MCL1 by Up-Regulating miR-107

Cell proliferation was performed as described previously with modifications. For the migration assay, the cells (2×10^5^ cells/well) were treated with taxol (0, 25, 50, and 100 µM) for 48 h, then trypsinized and resuspended in serum-free medium and 5×10^4^ cells were placed in the upper chamber of the well insert with 8 µm pore size polycarbonate membrane filter (Millipore). DMEM containing 20% fetal bovine serum was placed in the lower chamber. For the invasion assay, the experimental procedures are similar to the migration assay as described above, except the well insert was coated with 10 µL Matrigel (5 mg/mL; BD Biosciences, Bedford, MA). After incubation for 24 and 48 h at 37°C in the migration or invasion assay, respectively. Real-Time PCR was done and see described methods for more information. This experiment was performed twice independently.

### miRNA Target Prediction and Statistical Analysis

miRNA target sites were predicted using the software RNA22 online (https://cm.jefferson.edu/rna22v1.0/), TargetScan integrated with gNET algorithms. All experiments were performed at least in triplicate. Statistical significance was assessed using the Student t test. P <0.05 was considered significant.

## Results

### miR-107 Directly Targets MCL1

**Figure 1:**
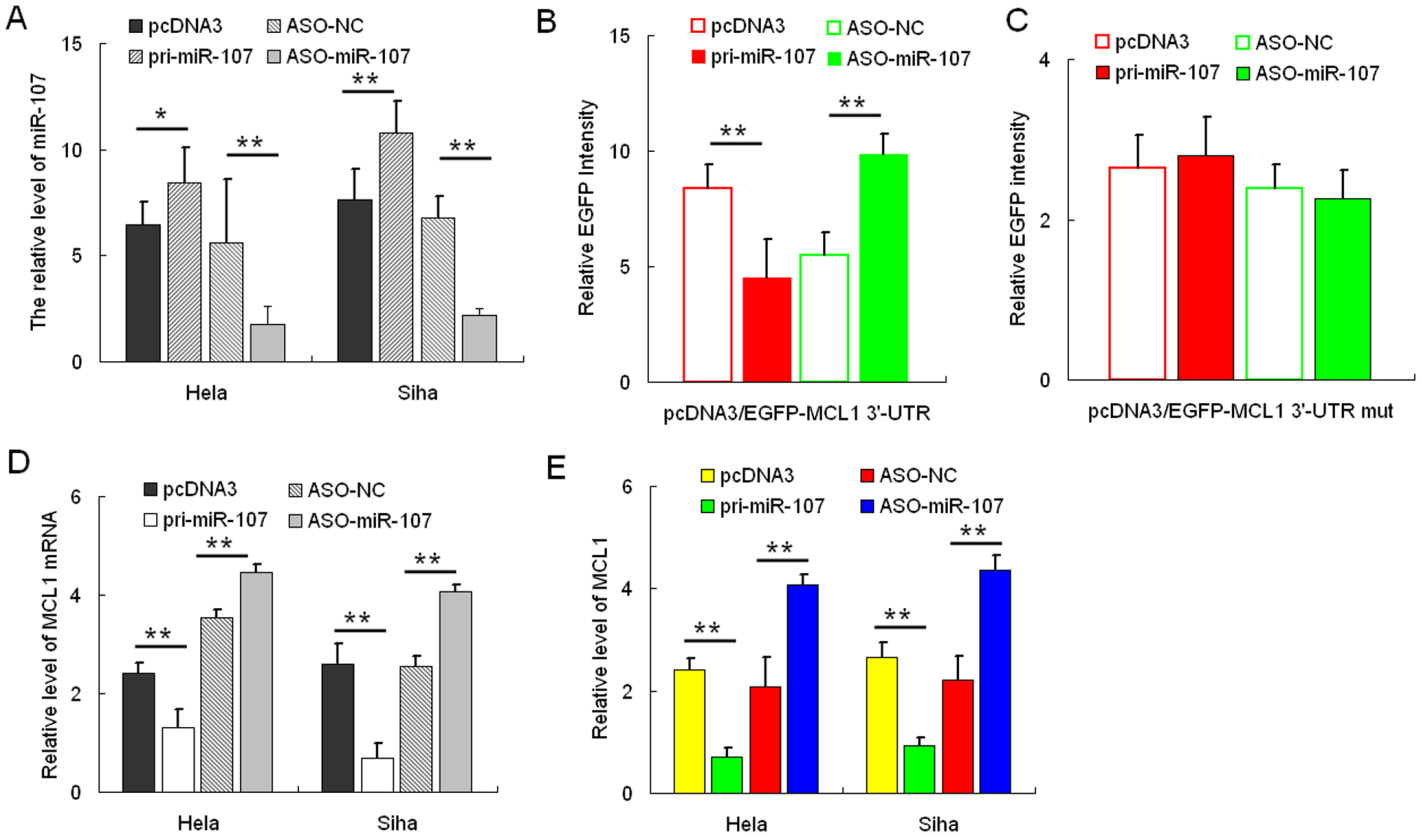
MCL1 is a direct target of miR-107. **A**, the expression level of miR-107 in HeLa and SiHa cells was significantly altered following transfection with either pri-miR-107 or ASO-miR-107 expression constructs as determined by qRT-PCR using U6 snRNA for normalization. **B**, the intensity of EGFP fluorescence in HeLa cells transfected with pri-miR-107 was decreased after 48 h and increased following transfection with ASO-miR-107. **C**, pri-miR-107 and ASO-miR-107 had no effect on the intensity of EGFP fluorescence in cells transfected with the 3′-UTR mutant vector. The mRNA (**D**) or protein (**E**) levels of MCL1 in HeLa and SiHa cells decreased or increased when compared with the control group when pri-miR-107 was overexpressed or blocked, respectively (*, *p*<0.05, **, *p*<0.005).

We firstly used the integrated computational algorithms of gNET method with TargetScan (see Fig. S1 in File S1) to identify gene-miRNA pairs significantly regulated in cervical cancer. We used this algorithm programs to predict miR-107 binding directly to 3′-UTR of MCL1. Next, the effect of miR-107 on MCL1 expression was validated by miR-107 gain and loss of functions. In both HeLa and SiHa cells, qRT-PCR was performed to validate the miR-107 over-expression construct or miR-107 ASO, with pcDNA3 or ASO-NC to be the respective controls (Fig. 1*A*). HeLa cells were co-transfected with the pcDNA3/EGFP-MCL1 3′-UTR report vector and pri-miR-107 or ASO-miR-107. As shown in Fig. 1*B*, the intensity of EGFP fluorescence in the pri-miR-107 group was significantly reduced, whereas that in the ASO-miR-107 group increased significantly at 48 h after transfection. To determine the function of the miR-107 binding site, we constructed an additional EGFP reporter vector containing the MCL1 3′-UTR with a mutant miR-107 binding site. As a result, neither overexpression nor blocking of miR-107 had any effect on the intensity of EGFP fluorescence in cells transfected with the 3′-UTR mutant vector (Fig. 1*C*). Together, these results demonstrated that miR-107 binds directly to the 3′-UTR of MCL1 to repress gene expression. Additionally, we determined whether miR-107 also suppresses endogenous MCL1 expression at the post-transcriptional level, we analyzed the effect of miR-107 on endogenous MCL1 mRNA and protein levels using qRT-PCR analysis and Western blotting, respectively. In HeLa cells, overexpression of miR-107 resulted in approximately 70% decrease in MCL1 mRNA levels (Fig. 1*D*) and about 50% decrease in protein expression (Fig. 1*E*). Furthermore, MCL1 mRNA and protein levels increased approximately 3- and 2-fold, respectively, in HeLa cells transfected with ASO-miR-107. We observed similar results in the SiHa cell line (Fig. 1, *D* and *E*). These data indicated that miR-107 negatively regulates endogenous MCL1 protein expression through mRNA degradation and translational repression.

### miR-107 Inhibits Migration and Invasion

**Figure 2:**
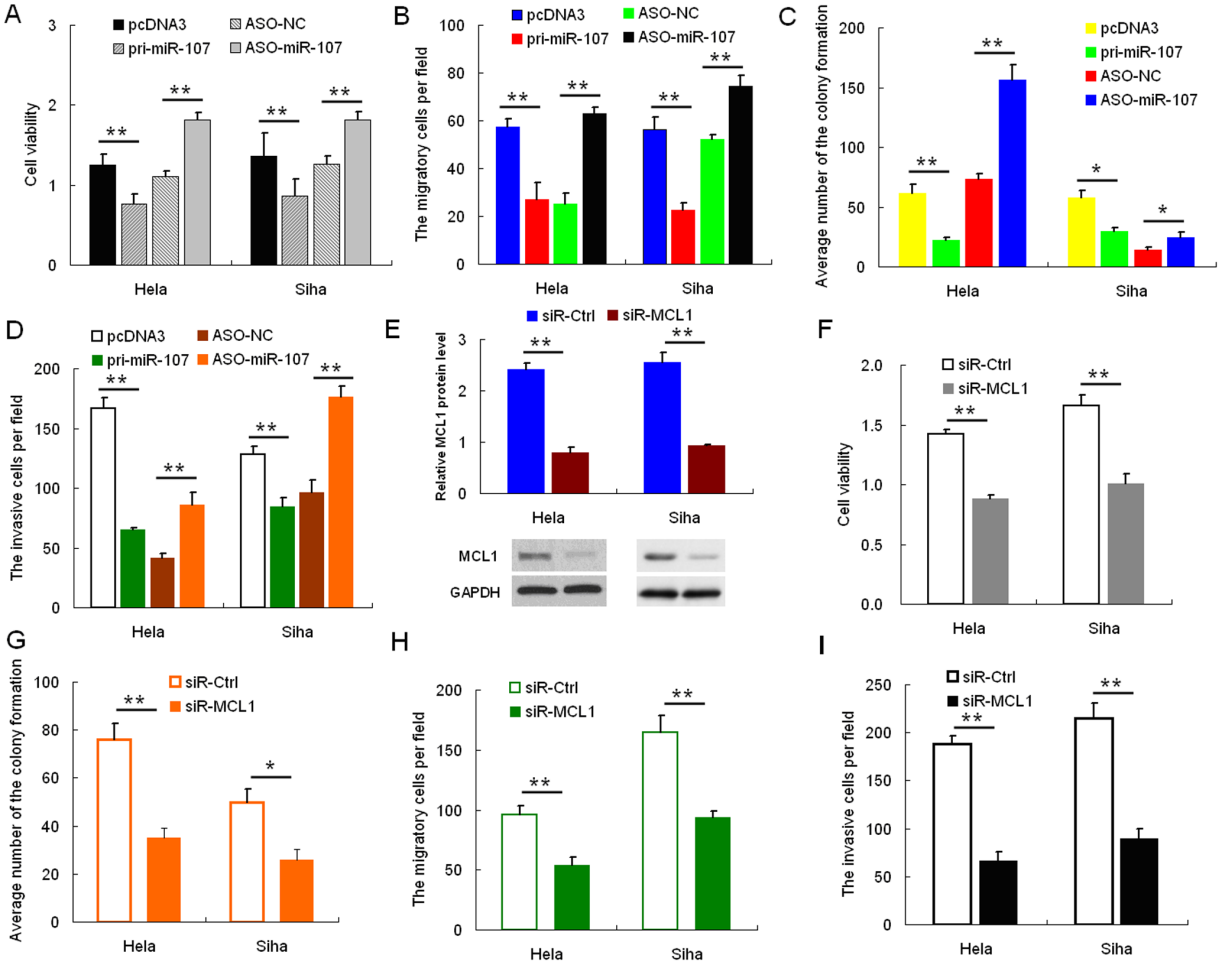
miR-107 represses cell proliferation and migration and invasiveness. Cervical cancer cells were transfected with either pri-miR-107 or ASO-miR-107. Cell viability was determined at 24, 48, and 72 h after seeding in 96-well plates using the MTT assay. *A*, the histogram shows the data at the time point of 48 h. All three data points showed a significant difference. *B* cervical cancer cells were transfected with pri-miR-107 or ASO-miR-107 and then seeded in 12-well plates. For the colony formation assay, the cells were stained with 2% crystal violet solution, and a representative image is shown. *C* and *D*, migration and invasion assays were performed with HeLa and SiHa cells transfected with either pri-miR-107 or ASO-miR-107. Representative images and randomly selected fields are shown. Knockdown of MCL1 suppresses proliferation, migration, and invasiveness of cervical cancer cells. *E*, MCL1 protein level was measured by Western blotting 48 h after transfection of siR-MCL1 into HeLa and SiHa cells. GAPDH was used as loading/transfer control (*Ctrl*) and for normalization of values. *F* and *G*, the effects of MCL1 knockdown on cell viability (*F*) were determined using the MTT assay, and cell proliferation was determined using the colony formation assay (*G*). *H* and *I*, changes in cell migration and invasiveness induced by siR-MCL1 were determined by migration and invasion assays. (*, p<0.05; **, p<0.005).

We performed MTT, colony formation, cell migration, and invasiveness assays using HeLa and SiHa cells transfected with either pri-miR-107 or ASO-miR-107 plasmids to determine the effects of miR-107 expression *in vitro*. MTT and colony formation assays demonstrated a statistically significant reduction in the cell viability and proliferation of the pri-miR-107-transfected cells relative to the control group, whereas ASO-miR-107 obviously increased these properties in HeLa cells (Fig. 2*A, B* and Fig. S2A in File S1). Transwell assay without Matrigel (Fig. 2*C* *and* Fig. S2B in File S1) demonstrated that miR-107 overexpression reduced migration in HeLa cells by 60%, and transfection of ASO-miR-107 increased migration by approximately two-fold compared with the control cells. Furthermore, overexpression of miR-107 resulted in a significant reduction in the invasive potential of HeLa cells when compared with control cells in Transwell assay with Matrigel, and cells transfected with ASO-miR-107 had a significantly increase in their invasive potential (Fig.2 *D* and Fig. S2C in File S1). Similar results were obtained with the SiHa cell line (Fig.2, *A*–*D*). These data suggest that miR-107 inhibits cell proliferation, migration, and invasiveness in HeLa and SiHa cells *in vitro*.

### Knockdown of MCL1 Inhibits Proliferation, Migration, and Invasion

The function of MCL1 in HeLa and SiHa cells was examined using RNA interference. HeLa and SiHa cells were transfected with siRNA targeting MCL1. Western blotting was used to evaluate the effect of siRNA on MCL1 protein inhibition. As shown in Fig. 2*E*, siR-MCL1 caused a statistically significant reduction of MCL1 protein levels, up to approximately 60% in HeLa cells and 50% in SiHa cells. Inhibition of MCL1 expression decreased viability (Fig. 2*F*) and colony formation (Fig. 2*G*) of cervical cancer cells when compared with control cells. Furthermore, knockdown of MCL1 expression resulted in a significant decrease in the rate of cell migration (Fig. 2*H*) and invasion (Fig. 2*I*). These findings demonstrated the effect of MCL1 knockdown on cell proliferation, migration, and invasion, which are consistent with the effect of miR-107 overexpression in both HeLa and SiHa cells.

### Restoration of MCL1 Counteracts Effects of miR-107 Expression

**Figure 3:**
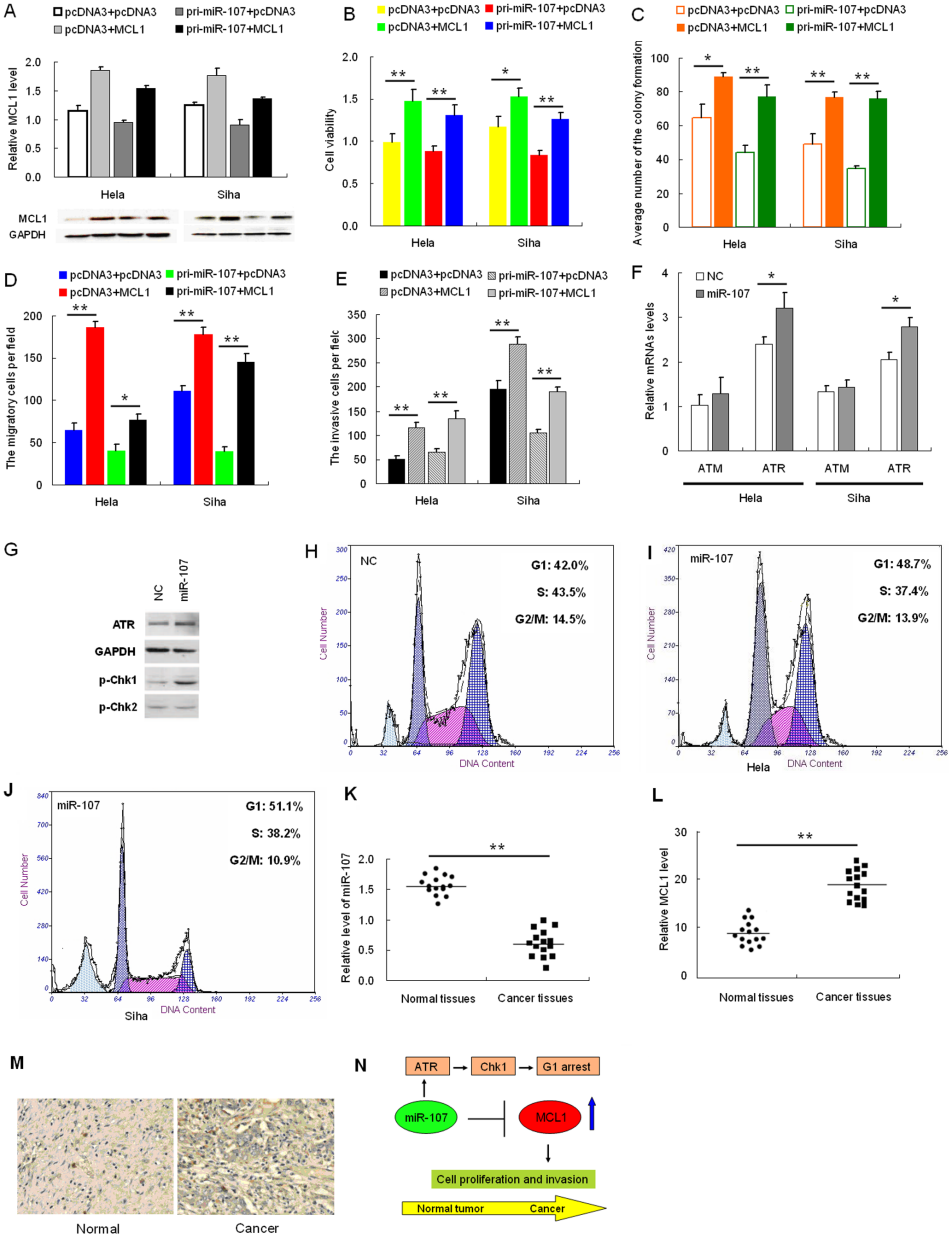
MCL1 rescues miR-107-induced cellular phenotypes in cervical cancer cells. *(A)*, cells were co-transfected with the pcDNA3/MCL1 vector, which did not contain the 3′-UTR of MCL1, with or without pri-miR-107 vector. (*B*–*D)*, at 48 h after transfection, the MCL1 protein level was measured by Western blotting. MTT assay (*B*), colony formation assay (*C*), transwell assays without Matrigel (*D*), or Transwell assays with Matrigel (*E*) were used to evaluate the cell viability, growth capacity, and potential for cell migration and invasion, respectively. (*F*) ATR and ATM mRNA levels were monitored by qRT-PC R at the indicated time points after miR-107transfection. GADPH was used for normalization. Data represent means ± standard error (SE) from three independent experiments. (*G*) Western blot assay revealed that after miR-107 overexpression, ATR was upregulated, then phosphorylation of Chk1 was increased, but phosphorylation of Chk2 was no difference. (H, I and J) Cell cycle distribution was evaluated in cells transfected with negative control, miR-107 or MCL1 by propidium iodide staining and flow cytometry 24 h after transfection. *N* and *O*, the relative expression of miR-107 (*K*) and MCL1 (*L*) in the 15 pairs, including cervical cancer tissues and matched normal tissues, was determined using qRT-PCR. *M*, expression of MCL1 in cervical cancer tissue and normal tissues by immunohistochemistry (*n* = 12). *N*, schematic representing miR-107-mediated regulation of MCL1 expression during cancer progression. Model of modified ATR/Chk1 pathway, in which miR-107 and MCL1 are involved. Data represent means ±SE from three independent experiments. Significance was determined by Student's t-test: *p<0.05, **p<0.005.

To confirm that the effects of miR-107 on the proliferation, migration, and invasiveness of HeLa and SiHa cells are mediated through MCL1, we constructed a pcDNA3/MCL1 vector containing the MCL1 ORF without the 3′-UTR to avoid the influence of miRNAs. Transfection of HeLa and SiHa cells with this MCL1 ORF expression construct reversed the negative effects of miR-107 on MCL1 protein levels (Fig. 3*A*). The inhibition of cell proliferation (Fig. 3*B*), colony formation (Fig. 3 *C* and Fig. S3A in File S1), migration (Fig.3 *D* and Fig. S3B in File S1), and invasiveness (Fig. 3*E* and Fig. S3C in File S1) caused by pri-miR-107 was abrogated in cells co-transfected with the pcDNA3/MCL1 vector. Over-expression of MCL1 countered the effect of miR-107 on cell proliferation, migration, and invasiveness of HeLa and SiHa cells.

### miR-107 Activates ATR/Chk1 Pathway

To verify that whether miR-107 could activate DNA damage pathways, we monitored the mRNA level of ATR and ATM (Fig. 3*F*). Gain of pri-miR-107 massively induced the expression of ATR but not ATM at mRNA level. Similar results were observed at protein level (Fig.3*G*). Compared with control, ATR protein was increased when primiR-107 was overexpressed. We also monitored the activation of Chk1 and Chk2, a downstream gene of ATR and ATM. As shown in Fig. 3*G*, Chk1 was dramatically phosphorylated after pri-miR-107 overexpression, however there was no significant change in phosphorylation of Chk2. Since activation of Chk1 usually results in G1 arrest, we tested the cell cycle profile with flow cytometry (Fig. 3 *H*, *I* and *J*). Correspondingly, the cells in S phase were decreased along with miR-107 overexpression whereas there was no significant change in G2/M phase. All these data suggest that increased miR-107 induces G1 arrest by activating ATR/Chk1 pathway.

### Expression of miR-107 and MCL1 in Cervical Cancer and Normal Tissues

To detect the expression of miR-107 in human cervical cancer tissues, the qRT-PCR assay was performed on fifteen paired samples of cervical cancer and adjacent normal tissues. miR-107 was expressed at a lower level (Fig. 3*K*), whereas MCL1 was expressed at a significantly higher level in the tumor tissues when compared with the corresponding normal tissues (Fig. 3*L*). In this setting, we utilized an immunohistochemistry assay to further detect the MCL1 expression level in human cervical cancer tissues and adjacent normal tissues. As shown in Fig. 3*M*, the expression levels of MCL1 were significantly higher than those in adjacent normal tissues, further supporting that miR-107 negatively regulates MCL1. The working model of the miR-107-MCL1 interaction during cancer progression was shown in Fig. 3*N*. This discovery provides insight into how MCL1 expression is regulated, and suggests therapeutic targets for rescuing the function of the MCL1 protein most commonly associated with human cervical cancer.

### Taxol Suppresses Migration and Invasion of Cervical Cancer

**Figure 4:**
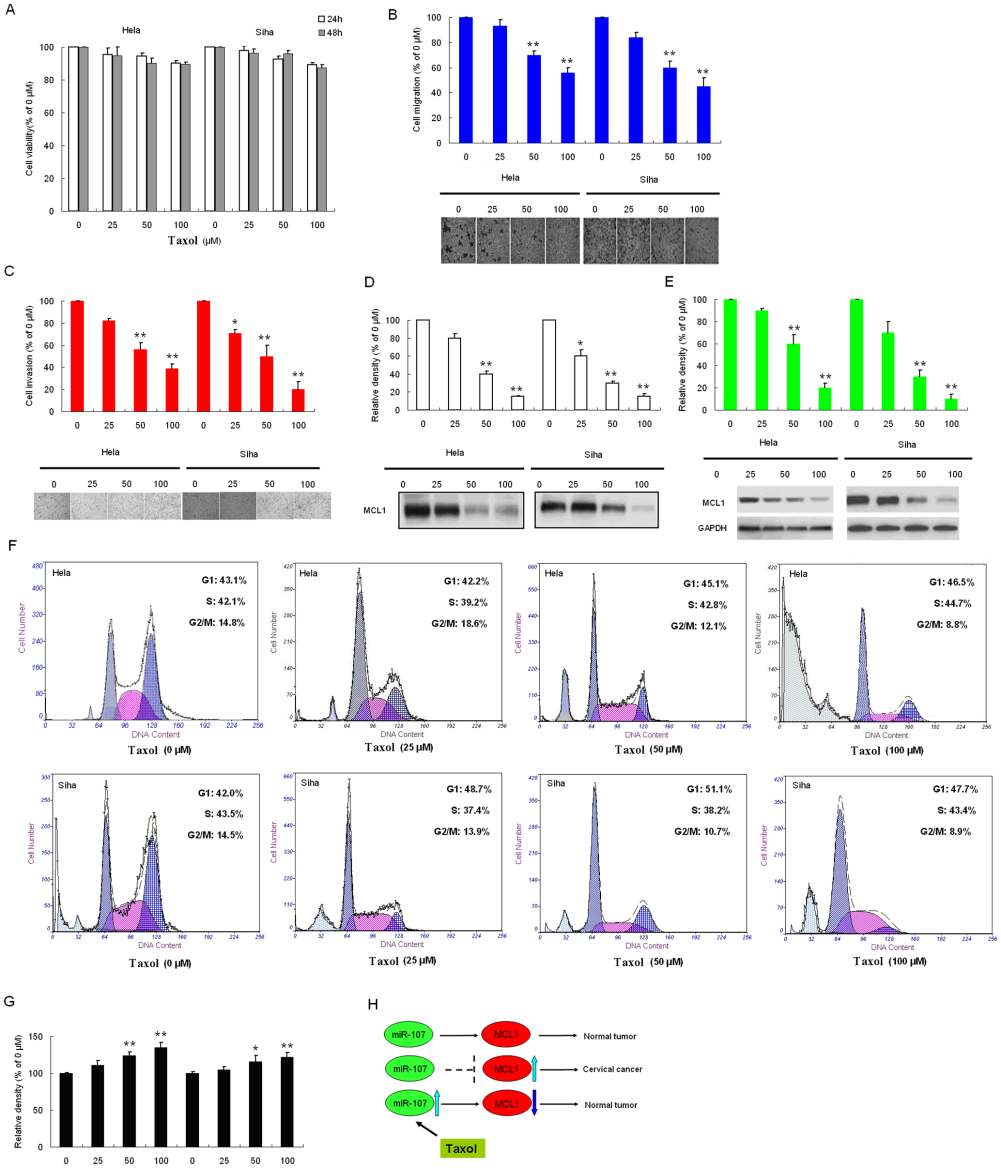
Effects of taxol on the viability, migration, and invasion in cervical cancer cells. (*A*)The chemical structure of taxol. (*B*) HeLa cells and SiHa cells were treated with increasing concentrations of taxol for 24 and 48 h. Cell viability was determined by MTT assay. The plot presents relative cell viability compared to untreated (0 µM) cells. HeLa and SiHa cells were treated with the indicated concentrations of taxol for 48 h. Subsequently, the migratory (*C*) and invasive (*D*) ability of cells after each treatment were determined, as described in material and methods. The bottom plots were the relative cell numbers comparing to that in untreated (0 µM) cells. Cells were treated with the indicated concentrations of taxol for 48 h. (*E*) the conditioned medium from each treatment was collected, and MCL1 activity was determined by casein zymography. (*F*) Cell lysate was applied to determine the protein levels of MCL1 by Western blotting. GAPDH was used as the internal control. (*G*) Apoptosis assay showed that taxol suppressed cell apoptosis and G1 arrest. (*H*) the relative expression of miR-107, (*I*) the working model of taxol regulates expression of MCL1 by down-regulating miR-107. Bars show the value as mean ±S.E. from three independent experiments. *, P<0.05; **, P<0.005, compared with the untreated cells.

In this study, we investigated the cytotoxicity of taxol by treating cervical cancer cells with various concentrations of taxol for 24 or 48 h. Results from the MTT assay showed that taxol was not significantly toxic to HeLa and SiHa (Fig. 4*A*) cells at the concentrations up to 100 µM for 24 to 48h. This range of concentrations was therefore applied in all subsequent experiments. Tumor cells detach from neighboring cells by releasing their intercellular junctions during metastasis, and the extracellular-matrix is proteolytically degraded to allow the migration and invasion of cancer cells. To investigate the effect of taxol on the malignancy of cervical cancer cells, the migration and invasion abilities of HeLa and SiHa cells were determined. In the cell migration assay, HeLa cells treated with 50 and 100 µM taxol showed a significant decrease in motility, respectively, and similar result was also observed in SiHa cells with inhibition (Fig. 4*B*). In the cell invasion assay, taxol was shown to reduce cell invasion in a concentration-dependent manner. At 50 µM, invasion was reduced by 46.12% and 61.24%, and at 100 µM, invasion was reduced by 50.04% and 80.11% in HeLa and SiHa cells, respectively (Fig. 4*C*). These results showed that taxol significantly inhibited the migration and invasion of cervical cancer cells under non-toxic concentrations.

### Taxol Inhibits the Expression of MCL1

To investigate the possible underlying anti-metastatic effect of taxol, the expression of MCL1 in cervical cancer cells treated with various concentrations of taxol were examined. As shown in the caseinolytic activity assay, MCL1 activity decreased in a dose-dependent manner after treatment with taxol. Quantification analysis indicated that MCL1 activity decreased by 20.2%, 60.1%, and 85.4% in HeLa cells, and by 40.5%, 70.8%, and 84.2% in SiHa cells when cells were treated with 25, 50, and 100 µM of taxol, respectively (Fig. 4*D*). Western blot analysis was performed to examine the protein expression of MCL1 in cervical cancer cells. Compared to the control group in both two cervical cancer lines tested, taxol inhibited the protein expression of MCL1 in a dose dependent manner (Fig. 4*E*). The results indicate that taxol inhibited both the activity and expression of MCL1 in cervical cancer cells. These findings suggest that the antimetastatic effect of taxol is related to the inhibition of MCL1 expression in cervical cancer cells. Apoptosis assays found that taxol suppressed cell apoptosis and G1 arrest (Fig. 4*F*). To further investigate whether the inhibitory effect of taxol on the expression of miR-107 in cervical cancer cells was at the level of mRNA expression, a semi-quantitative RT-PCR analysis was performed. As shown in Fig. 4*G*, after the treatment of taxol for 48 h, the relative level of miR-107 in both SiHa and HeLa cells also increased significantly in a dose-dependent manner, compared to the control group. The taxol-mediated change in the expression of MCL1, as indicated by the results from the Western blot analysis, suggesting that taxol might regulate miR-107 expression at transcription levels. The working model of taxol regulates expression of MCL1 by up-regulating miR-107 was shown in Fig. 4*H*.

## Discussion

miRNAs are a class of small noncoding RNAs that inhibit posttranscriptional expression of target mRNAs by binding to target sequences usually located in the 3′-UTR and may function as oncogenes or tumor suppressors. Several studies have revealed that miRNAs either prevent translation or promote the degradation of specific targets by binding to the 3′-UTRs of target mRNAs [19]–[21]. miR-107 is a member of the miR-15/107 superfamily and has been shown to be associated with several human cancer types. MCL1 was also found to be highly expressed in human malignancies, including HCC, and is discussed in terms of contributing to apoptosis resistance of HCC cells. Remarkably, MCL1 overexpression is associated with poor prognostic outcome in multiple myeloma, breast cancer, relapsing acute myeloid leukaemia and acute lymphocytic leukaemia, so the mechanisms that increase MCL1 levels are of paramount importance. Whether MCL1 up-regulation in human malignancies is causally linked to cervical cancer or a correlative finding that are not fully understood and still has not to be examined. Particularly, understanding the control of MCL1 is critical for the discovery and development of novel therapeutic agents for the treatment of cervical cancer.

Most cancer deaths occur as a result of metastasis rather than the original tumor; therefore, inhibiting cancer-cell metastasis is a crucial aspect of cancer prevention [22]. In this study, we investigated the role of miR-107 in cervical cancer and regulates proliferation and metastasis and invasion in cells by targeting MCL1. miRNA regulates target genes at both the mRNA and the protein levels, traditional methods simply using gene expression value for gene miRNA pair prediction may fail to pick up those genes with mainly protein level changes. To overcome this problem, we have taken advantage of the integrated computational algorithms of gNET method with TargetScan algorithms previously established and to predict that MCL1 is a candidate target of miR-107. We identified MCL1 as a target gene of miR-107, and found that miR-107 is down-regulated, whereas MCL1 is up-regulated in cervical cancer tissues when compared with adjacent normal tissues. miR-107 negatively regulates MCL1 at both mRNA and protein levels. Expression of an EGFP reporter containing the 3′-UTR of MCL1 was inhibited when miR-107 was overexpressed and activated when ASO-miR-107 was used. miR-107 suppresses and MCL1 promotes the proliferation, migration and invasiveness of HeLa and SiHa cells. Of note, we elucidated the underlying mechanisms by which taxol attenuates the migration and invasion of cervical cancer cells, possibly by activate the miR-107 and reducing the level of MCL1 expression, as well as having an anti-metastatic potential.

miR-107 reduced MCL1 levels in HeLa and SiHa cell lines, two cell lines with high MCL1 and low miR-107. Treatment with miR-107 significantly blocked cell proliferation, DNA replication, colony formation and invasion in HeLa and SiHa cells. Ectopic expression of miR-resistant MCL1 was sufficient to partially rescue the loss-of-function phenotype in miR-107-overexpressing HeLa and SiHa cells. Our results demonstrated that MCL1 was directly regulated by miR-107 and, moreover, suggested that miR-107 may be a potential anti-cancer therapeutic for cervical cancer. Taken together, these results suggested that in vitro miR-107 can bind to the 3′-UTR of the human MCL1 mRNA and inhibit protein synthesis by RNA degradation. Here, we identified that miR-107 was down-regulated in human cervical cancer. Interestingly, miR-107 is down-regulated in human breast cancer tissues [23], which is consistent with our results. One possible explanation is that biological molecules have different influences in different tumor cells. Moreover, we showed that MCL1 was negatively regulated by miR-107 at the posttranscriptional level, via a specific target site within the 3′-UTR by luciferase reporter assay. Furthermore, we replenished the ATR/Chk1 pathway by revealing the involvement of miRNAs. Subsequently, overexpression of miR-107 suppressed the expression of MCL1, which has been proved to be directly targeting on ATR/Chk1 pathway in our work. Accordingly, miR-107 upregulated ATR expression and activated ATR/Chk1 pathway but not ATM/Chk2 pathway. We revealed a fine tuning process of cells in response to DNA damage and replenished the ATR/Chk1 pathway, in which miR-107 played an important role by regulating the expression of MCL1.

Taxol has been shown to possess anti-tumor properties, and therefore poses special interest in the development of a therapeutic agent for cervical cancer. However, its effect on the anti-metastatic potential of cervical cancer cells remains unclear. The objective of this study was to investigate whether the migration and invasion of cervical cancer cells could be regulated by taxol. It provides insight into the way in which taxol modulates the aggressive phenotype. Fig. 4*H* shows the mechanism that taxol inhibits the migration and invasion in human cervical cancer cells. Suppression of cell migration and invasion by taxol via activate miR-107 expression, leading to down-regulation of MCL1 expression. Future studies on taxol may incorporate animal models to determine its efficacy in preventing migration and invasion in cervical cancer. Findings and observations from this study provide a crucial basis for further exploring the mechanisms of taxol and its potential for preventing tumor metastasis, and its possibility as an anticancer agent or an adjunct to current cancer therapies.

## Conclusions

miR-107 is expressed at a low level in cervical cancer when compared with normal cervical tissues, and overexpression of miR-107 inhibits cell growth and invasion. A new target gene of miR-107, MCL1, was found to be up-regulated in cervical cancer tissues. miR-107 suppressed the expression of MCL1, which has been proved to be directly targeting on ATR/Chk1 pathway. Therefore, the identification of miR-107 and its target gene, MCL1, in cervical cancer may help us to understand potential molecular mechanisms of tumorigenesis and may provide new prognostic markers for the management of cervical cancer. Together, our data reveals miR-107 as a potential biomarker of response to therapy in cervical cancer and highlights its potential as a therapeutic target. The expression and activity of MCL1 was significantly suppressed by taxol in a dose-dependent manner. We also elucidated the underlying mechanisms by which taxol attenuates the migration and invasion of cervical cancer. The taxol-mediated change in the expression of MCL1 suggests that taxol might regulate miR-107 expression at transcription levels. It provides strong evidence that using taxol as a therapeutic strategy against cervical cancer by inhibiting metastasis and invasion.

## Supporting Information Legends

File S1 Contains the following files: Fig. S1 the TargetScan integrated with gNET algorithms. Fig. S2 A, cervical cancer cells were transfected with pri-miR-107 or ASO-miR-107 and then seeded in 12-well plates. B and C, migration and invasion assays were performed with HeLa and SiHa cells transfected with either pri-miR-107 or ASO-miR-107. Fig. S3 MTT assay, colony formation assay (A), Transwell assays without Matrigel (B), or Transwell assays with Matrigel (C).
(DOC)
