# Supplementary material for: miR-107 Activates ATR/Chk1 Pathway and Suppress Cervical Cancer Invasion by Targeting MCL1
Source: PLoS One. 2014 Nov 11;9(11):e111860. doi: 10.1371/journal.pone.0111860 (PMC4227659; doi:10.1371/journal.pone.0111860)
Supplement: File S1 — Contains the following files: Fig. S1 the TargetScan integrated with gNET algorithms. Fig. S2 A, cervical cancer cells were transfected with pri-miR-107 or ASO-miR-107 and then seeded in 12-well plates. B and C, migration and invasion assays were performed with HeLa and SiHa cells transfected with either pri-miR-107 or ASO-miR-107. Fig. S3 MTT assay, colony formation assay (A), Transwell assays without Matrigel (B), or Transwell assays with Matrigel (C). (DOC) [file pone.0111860.s001.doc]

**
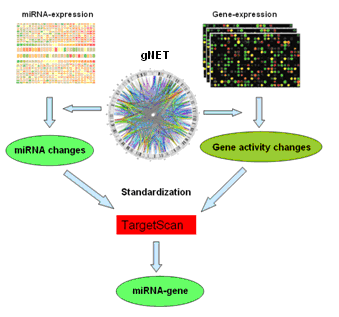
**

**Fig. S1** the TargetScan integrated with gNET algorithms.

**
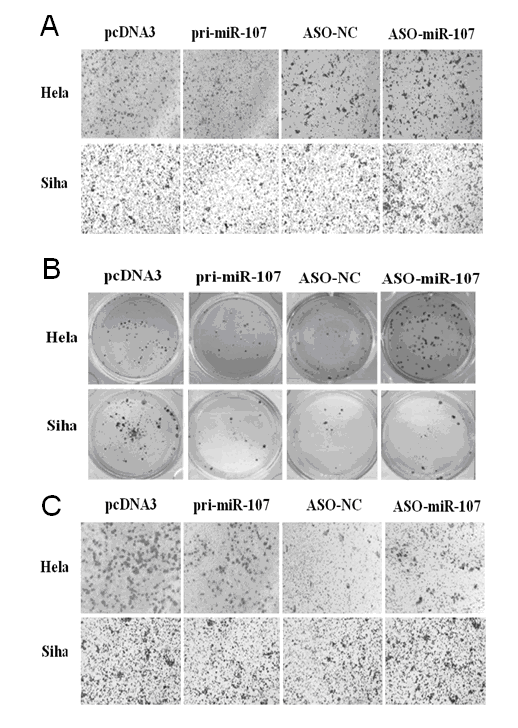
**

**Fig. S2** *A* cervical cancer cells were transfected with pri-miR-107 or ASO-miR-107 and then seeded in 12-well plates.

*B* and *C*, migration and invasion assays were performed with HeLa and SiHa cells transfected with either pri-miR-107 or ASO-miR-107.


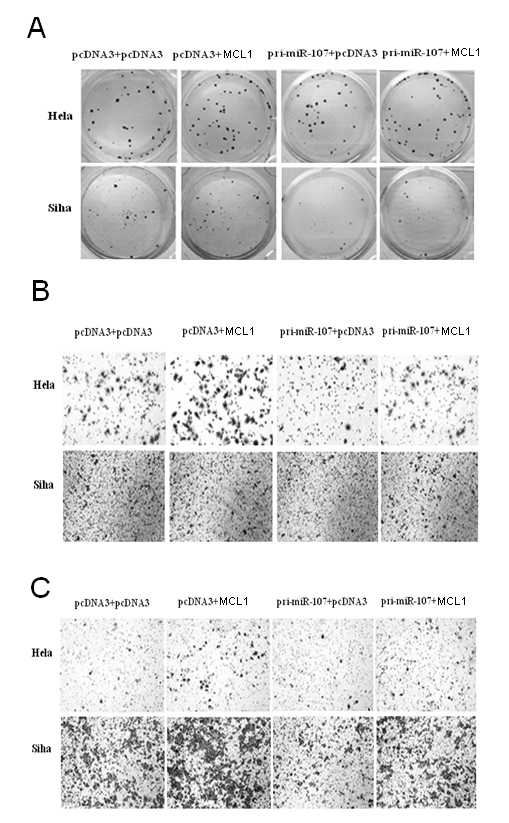


**Fig. S3** MTT assay, colony formation assay (*A*), Transwell assays without Matrigel (*B*), or Transwell assays with Matrigel (*C*)
